# Comprehensive Analysis of the Prognostic Implications and Biological Function of HDACs in Liver Hepatocellular Carcinoma

**DOI:** 10.7150/ijms.97169

**Published:** 2024-10-28

**Authors:** Zhaolei Cui, Chaoqiang Zheng, Yiqing You, Shijie He, Shan Jiang, Yan Chen, Yingying Lin, Zhenzhou Xiao

**Affiliations:** 1 Laboratory of Biochemistry and Molecular Biology Research, Department of Laboratory Medicine, Clinical Oncology School of Fujian Medical University, Fujian Cancer Hospital, Fuzhou, 350014, China.; 2 Department of Gynecology, Clinical Oncology School of Fujian Medical University, Fujian Cancer Hospital, Fuzhou, 350014, China.

**Keywords:** histone deacetylases (HDACs), CKD-581, liver hepatocellular carcinoma, prognostic model, cyclin

## Abstract

**Background:** The prognostic significance and biological functions of the histone deacetylases (HDACs) gene family in liver hepatocellular carcinoma (LIHC) have not been fully investigated.

**Methods:** Using Kaplan-Meier and Cox regression analysis, this study determined if HDAC genes were relevant for prognosis in LIHC. A regression model utilizing HDAC genes and the least absolute shrinkage and selection operator (LASSO) was created to foretell LIHC risk. A selective inhibitor of endogenous HDACs, CKD-581, was studied *in vitro* and *in vivo* to determine its effects on the development, invasion, migration, and proliferation of LIHC cell lines.

**Results:** Six HDACs were identified as correlating with the prognosis of LIHC. Overall survival (OS) was found to be shorter in individuals with higher risk scores when compared to those with lower risk scores, according to survival study. Natural killer cell infiltration was higher in individuals with lower risk ratings, which was mainly explained by the type II interferon (IFN) response. Limiting the activity of endogenous HDACs caused LIHC cell death by preventing their migration, invasion, and proliferation. *In vivo* studies confirmed that blocking HDAC expression inhibited tumor growth in mice. Further mechanistic studies showed that inhibition of HDACs expression elevates the protein levels of P21 and P27, and reduces those of cyclins A2, B1, D1 and E1.

**Conclusions:** The risk score prognostic model based on HDAC genes could provide a valuable prognostic biomarker for LIHC. CKD-581 prohibits LIHC progression via inhibiting the cell cycle signaling pathway. CKD-581 holds promise as a therapeutic agent for the clinical management of LIHC.

## Introduction

According to the most current cancer statistics from 2022, LIHC is the third most frequent disease worldwide and the sixth most common cancer overall. To add insult to injury, LIHC ranks second in male death rates and has a two- to thrice greater incidence in males than females in most locations 1. Because tumors grow so rapidly and are so hard to detect in their early stages, most patients with LIHC do not receive a diagnosis until the disease has progressed or metastasized. This highlights the tremendous clinical and societal importance of developing accurate techniques of prognosis assessment and targeted treatment approaches 2.

Critical to gene regulation and cellular metabolism is the histone deacetylase (HDAC) family of enzymes^3^. Within this family, there are four distinct classes that make up the 18 isoforms found in mammals: proteins belonging to class I Rpd3-like (HDAC1, HDAC2, HDAC3, and HDAC8), proteins belonging to class II Hda1-like (HDAC4, HDAC5, HDAC6, HDAC7, HDAC9, and HDAC10), proteins belonging to class III Sir2-like (SIRT1, SIRT2, SIRT3, SIRT4, SIRT5, SIRT6, and SIRT7), and proteins belonging to class IV (HDAC11) 4. HDACs deacetylate lysine residues on histone tails, which modulates gene expression. Given that epigenetic modifications are crucial in tumorigenesis and tumor progression, these enzymes can significantly influence cancer development 5. Previous research established that HDACs play a role in hematological malignancies 6, and more recent investigations have shown that glioma 7, renal clear cell carcinoma 8, breast cancer 9, and gastric cancer 10 all exhibit differential expression of HDACs. In addition, studies show that HDAC gene knockdown causes cell cycle arrest and apoptosis in cancers, demonstrating the significant correlation between HDAC expression, tumor cell proliferation, and tumor progression 11.

Studies on the HDAC gene family in LIHC are limited, most have focused on the functional role between only one or two HDAC genes and LIHC 12-16, and research on the prognostic feature of HDACs in LIHC is scarce. Yet to find out what role HDACs play in LIHC, we need to perform a thorough and methodical investigation of the whole HDAC gene family. Alteminostat (CKD-581; molecular weight, 492.61; molecular formula, C_27_H_36_N_6_O_3_) is an effective, specific inhibitor of HDACs, promoting the acetylation of histone H3 and microtubulin. Recent clinical study results showed that CKD-581 was safe, well-tolerated, and effective against cancer when used alone in treating trefractory lymphoma or multiple myeloma 17. In this study, our objective was to analyze the expression, mutations, function, and immune infiltration of the HDAC family of genes using online databases, assessing their potential oncogenic and prognostic values for LIHC. Concurrently, we corroborated our hypotheses by examining clinical samples. Utilizing the specific HDACs inhibitor CKD-581, we explored the biological functions and potential molecular mechanisms by which HDAC inhibition affects LIHC at the cellular level, and confirmed our results with *in vivo* experiments using CKD-581 in mice.

## Materials and methods

### Tissue samples

Approval No. SQ2015-049-01 was granted by the Clinical Research Ethics Committee of Fujian Cancer Hospital, and all subjects voluntarily gave their informed consent. We used three sets of LIHC tissue and neighboring non-tumor tissue samples that were taken during surgical excisions at Fujian Cancer Hospital between September 2022 and September 2023. All samples were shown to have LIHC by postoperative pathology. We treated one half of the samples in 4% paraformaldehyde and immersed the other half in paraffin for long-term preservation; the other half was kept in liquid nitrogen. Additionally, we acquired LIHC tissue cDNA microarrays (cDNA-HLivH090Su01) from Shanghai Xinchao Biotechnology Co. Ltd, which included data from 64 patients treated from April 2006 to May 2013, with follow-ups ranging from 2 to 9 years.

### Data collection and data processing

Expression, clinical, and single nucleotide polymorphism data were extracted from 374 LIHC cases and 50 controls in the Cancer Genome Atlas (TCGA) database. Additionally, clinical and ICGC expression data for LIHC patients were obtained. Furthermore, we obtained expression data for 33 common cancers and copy number variation (CNV) information for LIHC from the Xena database at UC Santa Cruz (UCSC). We next proceeded to analyze 18 HDAC genes in additional detail.

### Construction and validation of a prognostic model

Using information from the TCGA database, we created a model to forecast OS. The optimal genes for this prognostic model were selected based on a regression coefficient (coef) value in conjunction with genes that are both differentially expressed and associated to prognosis. The coef was determined by LASSO regression analysis, with replacement 1,000 times to compress the coefs of less impactful HDACs to 0, deeming them insignificant. The larger the absolute value of a coef, the greater its impact on the prognostic outcome.

The following is the formula for determining the risk score of each sample:

risk score = coef_1_ × gene_1_ expression + coef_2_ × gene_2_ expression + ... + coef_n_ × gene_n_ expression, where coef represents the corresponding HDAC coefficient, and gene expression denotes the respective HDAC expression level. Based on their median risk score, each patient was categorized as either high-risk or low-risk. To examine the OS variation among the groups, we used Kaplan-Meier survival curves. For patients undergoing TCGA and ICGC, we used receiver operating characteristic (ROC) curves to assess the accuracy of the prognosis for 1-, 2-, and 3-year OS. To categorize the risks, we performed principal component analysis. It was afterwards determined that the ICGC dataset had adequately verified the predictive model.

### Independent prognostic analysis and clinical correlation

In order to verify that the risk score is independent, we used both multivariate and univariate Cox regression analysis. We examined many clinical datasets using stratified analysis to see if risk ratings were associated with clinical features.

### Immune infiltration, tumor microenvironment, gene set enrichment, and drug sensitivity analysis

Immune cell scores and functions were quantified for each LIHC patient using a single-sample gene set enrichment analysis (ssGSEA), and these results were then compared across high-risk and low-risk categories 18. We calculated risk scores, immunological scores, and stromal scores for each category. We then performed enrichment analysis on the TCGA dataset to identify high-risk and low-risk groups. We used the Cell Miner database to retrieve information about gene expression and medication sensitivity. We then used P-values and correlation coefficients to investigate the link between gene expression and drug sensitivity.

### Reagents and animals

The Shanghai, China-based provider MCE supplied the CKD-581 medication. The C57BL/6J mice utilized in this research were procured from Shanghai Slake Experimental Animal Co., Ltd. The mice ranged in weight from 20 to 25 g, were eight weeks old, and were male. Procell Life Science&Technology Co.Ltd of Wuhan, China, was contacted in order to obtain Thepa1-6, Huh-7, and Bel7402. The study's mice were housed in a climate-controlled chamber that maintained a constant temperature, relative humidity, and light intensity. They had an endless supply of food and water. The study's method was green-lit by Fujian Medical University's Animal Ethics Center (ethics number: IACUC FJMU 2022-0723), and the mice were well-cared-for throughout.

### Cell proliferation, migration, and invasion assays

The proliferation of cells was measured using a Cell Counting Kit-8 from APExBIO in Houston, TX, USA. To summarize, with a density of 2 × 10^3^ cells per well, Bel7402, Huh-7, and Hepa1-6 cells were seeded into 96-well plates. We treated the cells with CKD-581 at doses of 1 µM and 10 µM after assigning five replicate wells to each group. Thermo Fisher Scientific's enzyme calibration was used to quantify the absorbance at 450 nm at 2, 4, 8, and 72 hours, respectively.

The cell migration assay was carried out by placing 2 × 10^4^ cells into one chamber of a 24-well plate (BIOLIF, Guangzhou, China) that did not have a membrane coating. The lower chamber contained 600 µL of complete medium, while this compartment had 200 µL of serum-free media. The cells were cultured at 37°C for 24 hours after being exposed to either 1 µM or 10 µM concentrations of CKD-581 in both chambers. Subsequently, cells were incubated with 4% paraformaldehyde on the membrane surface for 20 minutes, followed by the staining with 0.1% crystal violet. Afterwards, an inverted microscope (Olympus, Tokyo, Japan) was used to ascertain the cell count.

The cell invasion assay was conducted by coating the bottom of the upper chamber with 80 µL of matrix gel (Corning Inc., Corning, NY, USA). The subsequent steps were identical to those used in the migration assay.

### Plate cloning assay

A total of 1 × 10^3^ cells per well of six-well plates containing complete medium were used to culture the logarithmic growth phase cells. Subsequently, CKD-581 was introduced to the cells at concentrations of 1 µM and 10 µM. Over the course of three weeks, the medium was swapped out every three days. Upon becoming visible, the clones were rinsed three times with phosphate-buffered saline (PBS, pH 7.4). After that, they were incubated in 4% paraformaldehyde for 30 minutes, dyed crystal violet, and then rinsed. The clones were subsequently photographed in order to count them.

### Apoptosis assay

In order to get 1 × 10^6^ cells/mL, the cells were first collected, rinsed twice with 4°C precooled PBS, spun in a centrifuge to collect liquid above the cells, and then resuspended in 1×Binding Buffer. After that, each cell tube was supplemented with 5 µL of Annexin V-PE and 5 µL of 7-AAD. After a gentle mixing, the tubes were incubated in the dark at 25°C for 15 minutes. The reaction was stopped by adding 400 μL of 1× Binding Buffer, and it was tested on a CytoFlex machine (Beckman Coulter Life Sciences, USA) within an hour.

### Real-time quantitative PCR (RT-qPCR) analysis

The cells or tissues that were exposed to CKD-581 (1 µM, 10 µM) for 24 hours had their total RNA harvested, and a reverse transcription kit from Roche (Basel, Switzerland) was used to generate complementary DNA. The PCR primers listed in Table 1 were acquired from Sunya in Fuzhou, China. An RT-qPCR assay using a SYBR Green kit from Roche was run on an ABI 7500 System (ABI, Vernon, CA, USA). To find the relative gene expression, the formula (2^-ΔΔct^) was utilized, and to find ΔCt, the Ct value of each target gene was subtracted from the Ct value of glyceraldehyde-3-phosphate dehydrogenase (GAPDH).

### Immunoblotting analysis

The cells and tissues that were exposed to CKD-581 (1 µM, 10 µM) for a period of 24 hours were lysed using RIPA lysis buffer (EpiZyme, China, PC101), which includes inhibitors for protease and phosphatase. Bichincinchoninic acid (BCA) protein kits (EpiZyme, China, ZJ102) were used to ascertain protein quantities. Prior to being transferred to PVDF membranes, twenty micrograms of protein were electrophoresed on sodium dodecyl sulfate-polyacrylamide gels at 10% or 15% concentrations (SDS-PAGE). Following an hour of blocking with 1× Protein Free Fast Blocking Solution (EpiZyme, China, PS108) at room temperature (18-25°C), the membranes were incubated with primary antibodies overnight at 4°C. Antibodies used included: P21 (AP021), P27 (AP027), cyclin A2 (AF2524), cyclin B1 (AF6627), cyclin D1 (AF1183) from Beyotime Biotechnology Co. Ltd (Shanghai, China), and cyclin E1 (11554-1-AP) from Proteintech Group (Wuhan, China). For P21, the primary antibody dilution was 1:500, whereas for P27 and cyclins A2, B1, D1, and E1, it was 1:1000. The membranes were immersed in secondary antibody for one hour at room temperature after three washes with TBS-Tween (TBST) buffer. The next step was to visualize the results using enhanced chemiluminescence reagents.

### Immunohistochemistry

From the fixed tissue samples, 4 µm thick sections were cut. Following dewaxing, rehydration, and antigen recovery, slices were incubated with primary antibodies at 4°C for the night. Antibodies including HDAC1 (GB11333-100), HDAC4 (GB115576-100), and SIRT7 (GB11355-100) were sourced from Wuhan Sevier Biotechnology Co.; HDAC5 (29342-1-AP), HDAC11 (67949-1-Ig), and SIRT6 (13572-1-AP) were obtained from Wuhan Sanying Biotechnology Co. The next step was a PBS wash, followed by incubation with a secondary antibody (Zhongsui Jinqiao, Beijing, China) at 37°C for 60 minutes, followed by another wash. Color development was achieved using DAB for 2 minutes, followed by hematoxylin restaining, and microscopy images were captured.

### Immunofluorescence analysis

After cell digestion, 5 × 10^4^ cells/well were plated on a 24-well plate, subjected to CKD-581 treatment, and then left to incubate overnight at 37°C with 5% CO_2_. Using 4% paraformaldehyde, the cells were adhered on creep plates after three washes with PBS. They become permeable after 20 minutes. After that, fluorescent primary antibodies were added to the cells and left to overnight at 4°C. For P21 and P27 as well as cyclins A2, B1, D1, and E1, we utilized the identical primary antibodies diluted to a 1:200 ratio as in the immunoblotting study. The next step was to let the cells sit at room temperature for an hour while a secondary fluorescent antibody was added. After that, DAPI was applied in the dark for 5 minutes. Images were captured after sealing.

### *In vivo* tumor xenograft analysis

Hepa1-6 cells (1.75 × 10^6^ cells in 100 µL saline) were subcutaneously injected into the right forelimb of eight-week-old C57BL/6J mice (n = 21). Once over 60% of mice developed subcutaneous tumors up to 50 mm^3^, they were divided into two groups: a control group (n = 11) and a treatment group (n = 10; 30 mg/kg). The treatment group received intraperitoneal injections of CKD-581 in 0.9% saline once weekly for two weeks, while the control group received no treatment. Using Vernier calipers, the size of the tumors were measured, and the mice were weighed three times weekly. The formula for tumor volume was (length) × (width)^2^ × π/6. A two-week interval followed the intraperitoneal injection of 2% pentobarbital sodium (100 mg/kg) that put the mice to sleep. Checks on vital signs were performed ten minutes later. Alanine aminotransferase (ALT) and aspartate aminotransferase (AST) serum levels were tested using a kit (Nanjing Jiancheng Corp. Nanjing, China) following centrifugation of five separate 0.1 ml blood samples taken from the eye. Tumor, liver, and spleen were collected, and no adverse effects such as ulceration or suppuration of tumors were observed during the experiment.

### Statistical analysis

Our statistical methods included two-sample t-tests for pairwise comparisons and one-way ANOVA and Kruskal-Wallis tests for multiple group comparisons. When the *P*<0.05, statistical significance was established (*P*<0.05, *P*<0.01, *P*<0.001). This is how the data is displayed: mean ± standard deviation (SD). Software packages such as R (4.1.3), PERL (5.30.1), SPSS (25), and GraphPad Prism (8) were utilized.

## Results

### Pan-cancer analysis and mutational mapping of the HDAC family of genes

**Figure 1:**
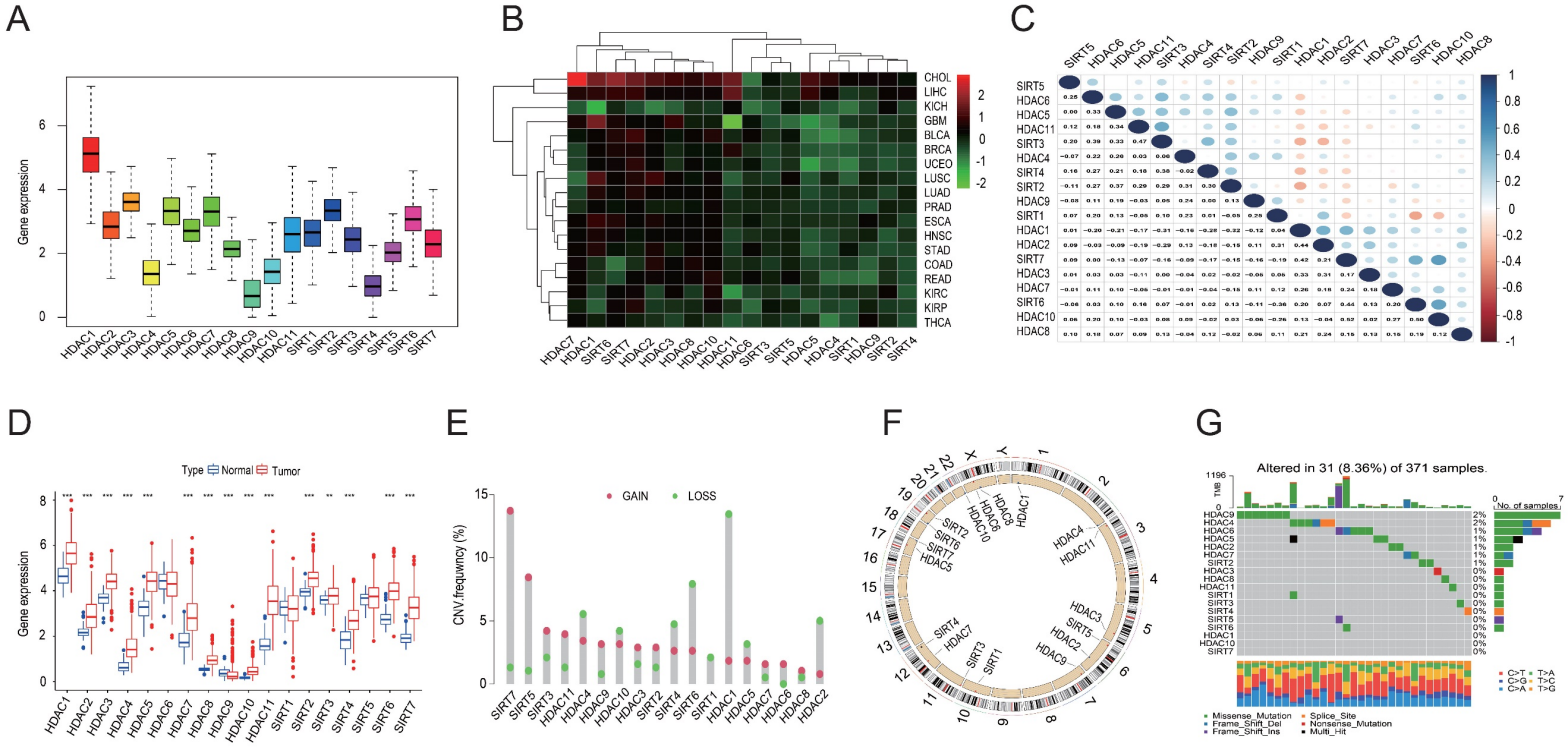
Pan-cancer and mutation analyses of the HDAC gene family. (A) Average expression levels of HDAC genes across 33 tumor types in TCGA. (B) Differential expression of HDAC genes across various tumor types, with red denoting up-regulation and green indicating down-regulation. (C) Correlation analysis of HDAC genes employing Spearman's correlation coefficient. (D) Comparative expression of HDAC genes in tumor versus normal tissues. (E, F) Copy number variations of HDAC genes in LIHC tissues and their chromosomal localization. (G) Somatic mutation analysis of HDAC genes. **P<0.01, ***P<0.001

Expression data for 33 tumors were obtained from the UCSC Xena database, leading to pan-cancer analyses. Subsequently, 18 HDAC genes were analyzed further. Differential analysis revealed high overall expression of HDAC genes in all tumor samples. *HDAC1* was the most highly expressed, while *HDAC4*, *HDAC9*, and *SIRT4* were expressed at relatively lower levels (Figures 1A and 1B). With the exception of *HDAC1*, *SIRT6*, and *SIRT7*, most HDAC genes were down-regulated in various tumor types. Pearson correlation analysis indicated correlations between certain HDAC genes, such as *HDAC10* and *SIRT7* (r = 0.52), *HDAC10* and *SIRT6* (r = 0.5), *HDAC11* and *SIRT3* (r = 0.47), and *SIRT1* and *SIRT6* (r = -0.36) (Figure 1C). Furthermore, with the exception of *HDAC9*, the majority of HDAC family genes were substantially elevated in tumor tissues when comparing mRNA expression in LIHC tissues to normal tissues (Figure 1D). CNV analysis in LIHC indicated increased CNV in *SIRT7*, *SIRT5*, and *SIRT3*, and decreased CNV in *HDAC1*, *SIRT6*, and *HDAC2* (Figure 1E). Chromosomal locations of HDAC family gene copy number variants were displayed (Figure 1F), with mutations found in 31 of the 371 LIHC samples (8.36%). The highest mutation rates were observed in *HDAC9* and *HDAC4* (2%), followed by *HDAC6*, *HDAC5*, *HDAC2*, *HDAC7*, and *SIRT2* (1% each) (Figure 1G).

### Screening of prognosis-related HDAC genes in LIHC and construction of a risk score prognostic model

**Figure 2:**
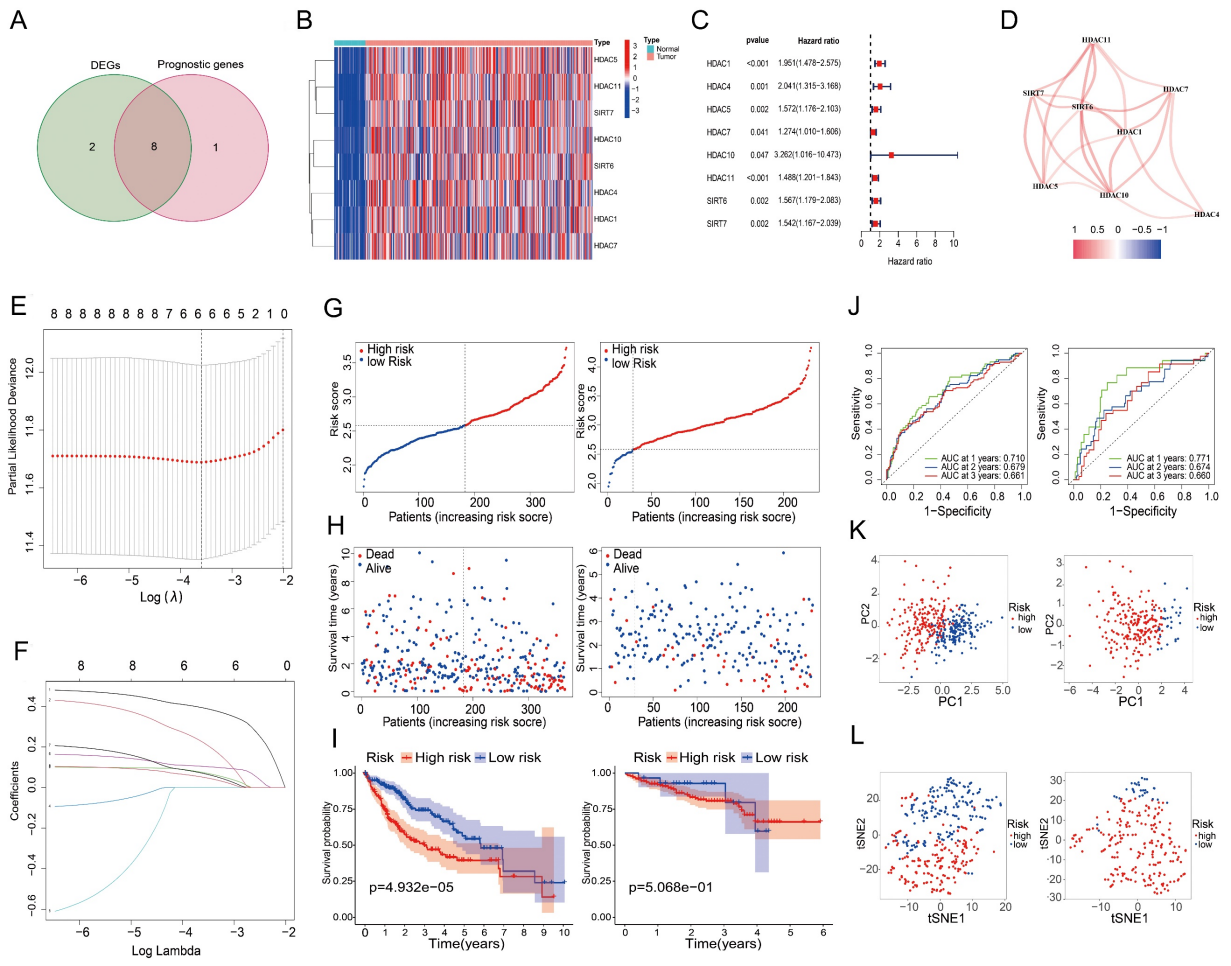
Prognostic screening of HDAC genes in the TCGA dataset. (A) Venn diagram identifying genes at the intersection of differentially expressed and prognosis-related genes. (B) Expression levels of eight intersecting genes in LIHC versus normal tissues. (C) Forest plot depicting the association between the expression of the eight intersecting genes and OS. (D) Network of correlations among intersecting genes. (E, F) Validation of the prognostic model using LASSO regression to determine the requisite number of genes. Panel E displays the cross-validation curve, which shows how the model's mean squared error varies with changes in Log (λ), indicating that reducing the candidate HDACs to 6 provides the optimal prognostic model; Panel F represents the regression coefficient path diagram, which demonstrates the process of reducing variable numbers and adjusting coefficients in the LASSO regression model. (G) High and low-risk scores, (H) survival status of patients, (I) survival curves, and (J) ROC curves for the test and validation cohorts. (K, L) Principal component and t-SNE analysis of the test and validation cohorts.

An investigation of 18 HDAC genes was conducted to determine the potential involvement of HDACs in the prognosis of LIHC. The analysis identified 10 genes as differentially expressed (*P* < 0.05) (Table 2). Furthermore, 9 of these genes were found to have prognostic value in the univariate Cox regression analysis (*P* < 0.05) (Table 3). Of these, eight genes were found at the intersection of differentially expressed and prognosis-related genes (Figure 2A). Heat maps confirmed that all intersecting genes were upregulated in LIHC tissues (Figure 2B). Univariate Cox regression of the intersecting genes demonstrated a negative correlation between their expression and survival rates in LIHC patients (Figure 2C). Correlations among the intersecting genes are depicted in Figure 2D. After excluding patients with a survival duration of 0 months, LASSO regression was utilized to analyze the expression profiles of the eight intersecting genes, resulting in the identification of six genes most suitable for constructing the prognostic model (Figures 2E and 2F), thereby eliminating overfitting genes. Risk scores were calculated as follows using the 6 HDAC genes:

Risk score = 0.392 × ExpHDAC1 + 0.221 × ExpHDAC4 + 0.074 × ExpHDAC5 + 0.104 × ExpHDAC11 + 0.069 × ExpSIRT6 + 0.05 × ExpSIRT7

To validate the model, we used the ICGC dataset, and to test it, we used the TCGA dataset. Figure 2G (left) shows the results of the analysis that classified the test group patients as either high-risk or low-risk based on their median risk scores. Figure 2H (left) shows that compared to low-risk patients, high-risk patients died earlier. Based on the Kaplan-Meier curves, the high-risk group had a significantly lower OS (Figure 2I, left). Based on the ROC curves (Figure 2J, left), the 1-year OS, 2-year OS, and 3-year OS AUCs were 0.710, 0.679, and 0.661, respectively. Principal component analysis and t-SNE analysis effectively distinguished patients into two distinct subgroups within the risk categories (Figure 2K and 2L, left). The same analyses were performed on the validation group data, with results paralleling those of the test group, affirming the predictive reliability of the constructed risk score prognostic model (Figures 2G to 2L, right).

### Analysis of LIHC prognosis and tumor microenvironment

**Figure 3:**
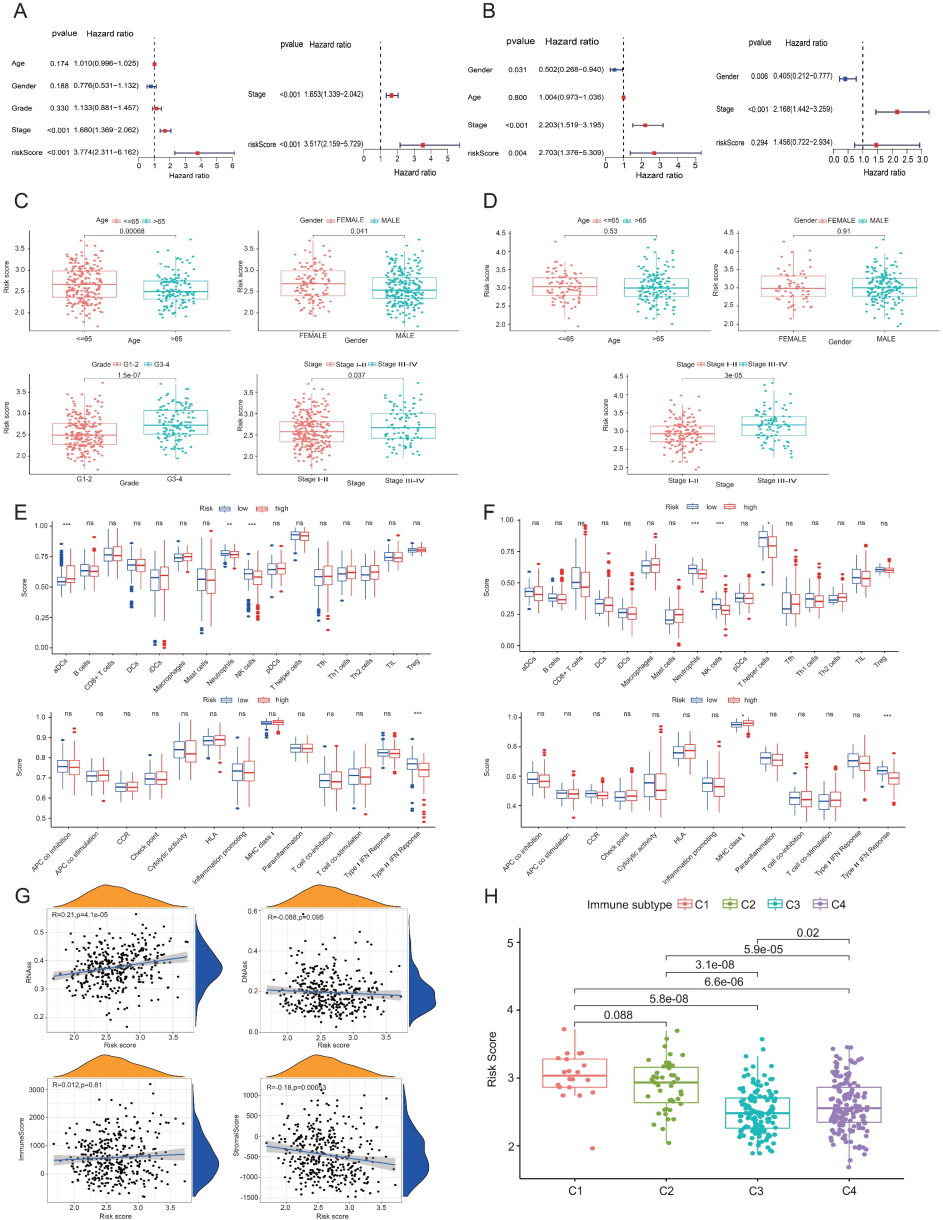
Prognostic and tumor microenvironment analysis in the test and validation groups. (A, B) Univariate and multivariate Cox regression analyses of prognostic factors in the test and validation groups. (C, D) Correlations between clinical characteristics and risk scores in LIHC patients within the test and validation cohorts. (E, F) ssGSEA scores for immune cells and immune-related pathways in both groups. (G) Associations between risk score and various scores (RNAss, DNAss, stromal, and immune). (H) Risk scores across different immune infiltration subtypes. ns, not significant; *P<0.05, **P<0.01, ***P<0.001

Figures 3A and 3B show the findings of the test and validation groups' univariate and multivariate Cox regression analysis for OS, respectively. Because of its strong correlation with both survival duration and clinical outcomes, the risk score seems to function as a standalone predictive variable in LIHC patients. When examining the correlation between the risk score and clinical characteristics in the test group, it was shown that patients with stage III-IV LIHC had much higher risk scores than those with stage I-II (*P* < 0.05) (Figure 3C). Figure 3D shows similar findings in the validation group. To explore possible alterations in immune responses, we used the ssGSEA approach to compare the enrichment scores of thirteen immune-related pathways and sixteen immune cells between the low-risk and high-risk cases grouped based on the 6 HDAC genes. Increases in neutrophil and natural killer (NK) cell counts, as well as scores of type II IFN responses, were observed in the low-risk group (Figures 3E and 3F).

The findings across the test and validation groups showed high consistency (Figures 3E and 3F). An RNA stem cell score (RNAss) based on messenger RNA expression and a DNA stem cell score (DNAss) based on DNA methylation patterns were utilized to determine tumor stem cell scores 19. Two measures, the immune score and the matrix score, were utilized to assess the tumor's immunological microenvironment. The risk score (based on the 6 HDAC genes) has a positive correlation (*P* < 0.01) with RNAss and a negative correlation (*P* < 0.01) with matrix score, as shown in Figure 3G. A considerable correlation between C1 and high-risk scores and C3 and low-risk scores was seen when using TCGA-LIHC data for immune infiltration analysis in LIHC (Figure 3H).

### GSEA and drug sensitivity analysis

**Figure 4:**
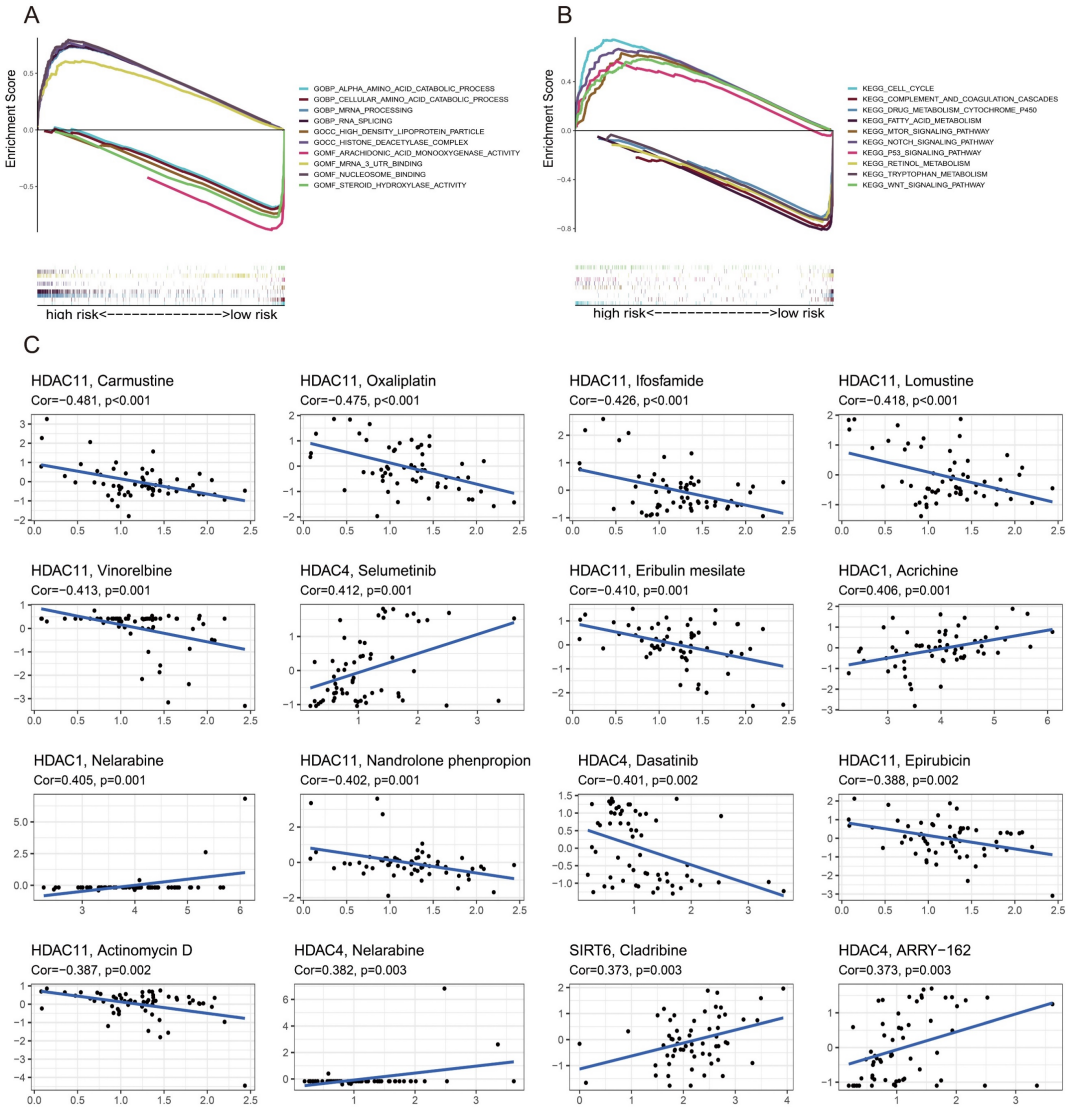
GSEA and drug sensitivity analysis. (A) GO and KEGG (B). (C) Analyses linking prognostic gene expression to drug sensitivity.

In order to compare the two groups based on risk, GSEA software was used to perform functional and Kyoto Encyclopedia of Genes and Genomes (KEGG) pathway enrichment studies, as well as Gene Ontology (GO) analyses. Figure 4A shows the results of the GO enrichment analysis, which determined that the high-risk group was connected with RNA splicing, the HDAC complex, and mRNA 3' untranslated regions (UTR) binding. According to KEGG enrichment analysis, the low-risk group was associated with pathways involving tryptophan metabolism, fatty acid metabolism, and retinol metabolism (Figure 4B), whereas the high-risk group was linked to the cell cycle, Notch signaling route, and P53 signaling pathway. The correlation between drug sensitivity and gene expression levels was examined in a study that found that higher levels of *HDAC11* were associated with increased resistance to carmustine, oxaliplatin, ifosfamide, lomustine, vinorelbine, eribulin mesylate, nandrolone phenylpropionate, actinomycin D, and epirubicin. Figure 4C shows that, on the other hand, selumetinib, nelarabine, and bimetinib sensitivity increased with increasing *HDAC4* expression levels.

### Validation of clinical samples

**Figure 5:**
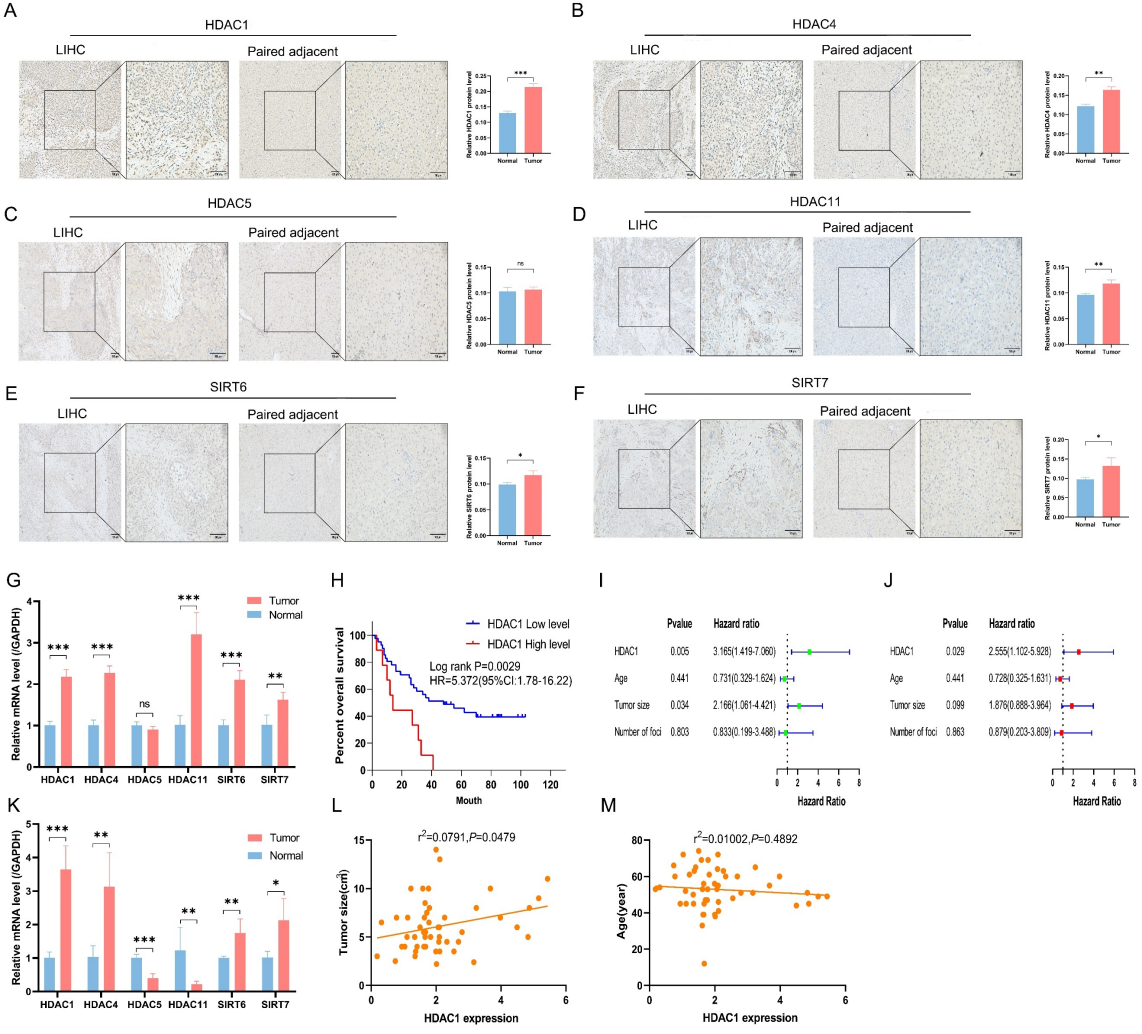
Validation of clinical samples. (A-F) Immunohistochemical staining of six HDACs across 6 samples of LIHC tissue (A, C, E; n=3) and paired paracancerous tissue (B, D, F; n=3). (G) The mRNA expression levels of six model genes in human LIHC tissues versus paracancerous tissues. (H) Kaplan-Meier analysis of OS based on HDAC1 expression. (I, J) Univariate and multivariate Cox analyses. (K) The mRNA expression levels of six model genes in mouse tumor and normal liver tissues. (L) Correlation analysis between mRNA levels of HDAC1 and tumor size. (M) Correlation analysis of mRNA levels of HDAC1 with age. LIHC, liver hepatocellular carcinoma; ns, not significant; *P < 0.05, **P < 0.01, ***P < 0.001

Apart from HDAC5, the other five HDACs displayed higher staining and more positive regions in cancer tissues, according to immunohistochemistry results from 3 sets of LIHC tissues and corresponding normal paracancerous tissues (Figures 5A to 5F). RT-PCR results supported this observation (Figures 5G and 5K). From the preliminary prognostic model, it was noted that HDAC1 was the most highly expressed among the six model genes and contributed the most to the model's weight. Thus, HDAC1 expression in 64 LIHC cases was assessed by RT-PCR. High HDAC1 expression was associated with a decreased OS after patients with missing clinical data were excluded (Figure 5H). High HDAC1 expression was linked to a poor prognosis in both univariate and multivariate Cox analyses, indicating that it could be a standalone prognostic factor in LIHC (Figures 5I and 5J). Furthermore, correlation analyses between HDAC1 expression, tumor size, and age showed a positive correlation between HDAC1 mRNA levels and tumor size (*P*<0.05) (Figures 5L and 5M).

### Effect of endogenous HDAC expression inhibition on the biological functions of LIHC cell lines

**Figure 6:**
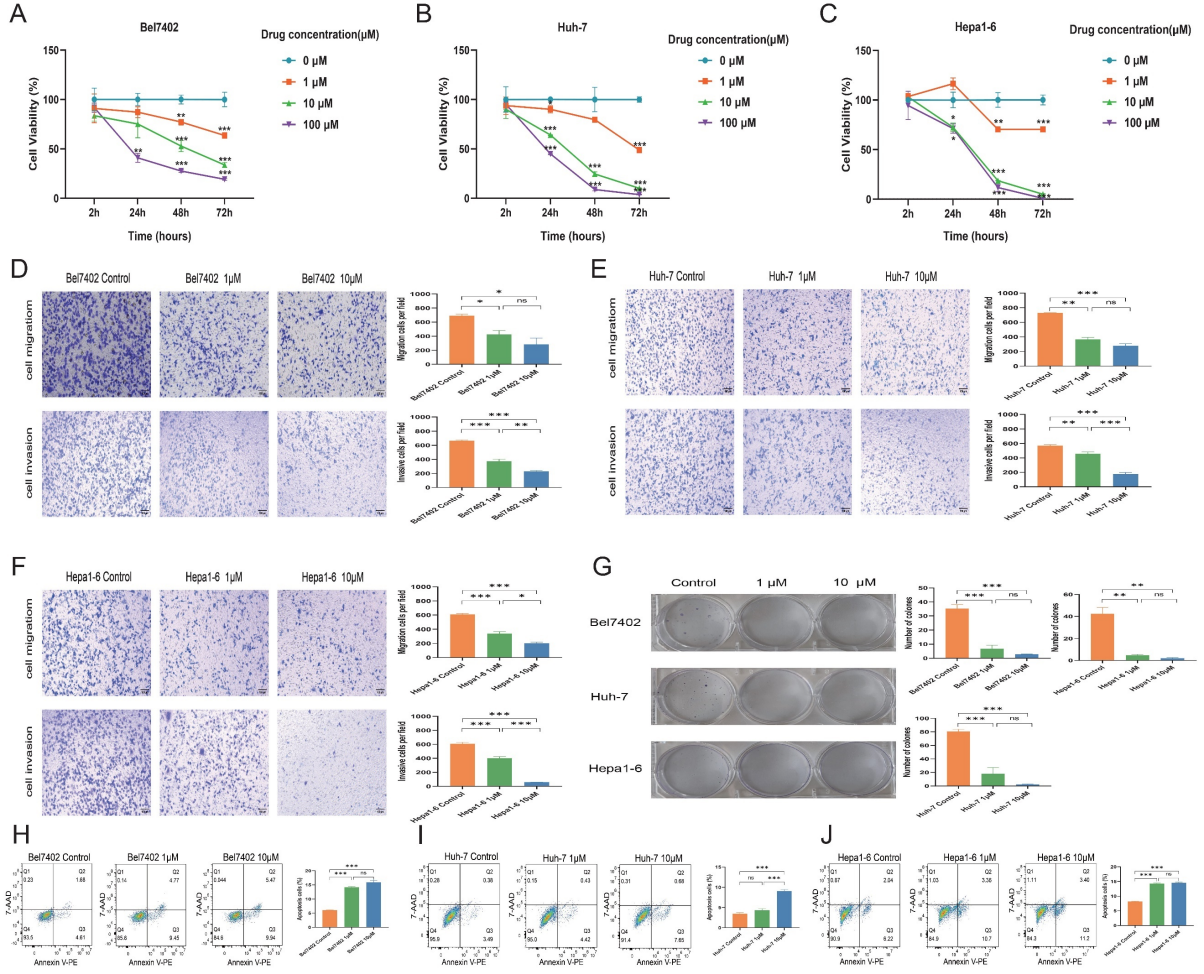
CKD-581 inhibits the proliferation, migration, and invasion of LIHC cells. CKD-581 significantly reduces the proliferation of (A) Bel7402 cells, (B) Huh-7 cells, and (C) Hepa1-6 cells. IC_50_ was calculated at 24h after treated with CKD-581. (D-F) Transwell assay for evaluating the migration and invasion capabilities of LIHC cells. (G) Clonogenic assay for LIHC cells. LIHC, liver hepatocellular carcinoma. (H-J) Flow cytometry analysis of apoptosis in Bel7402, Huh-7, and Hepa1-6 cells at 12h after treated with CKD-581. ns, not significant; *P < 0.05, **P < 0.01, ***P < 0.001

Bel7402 (IC_50_ = 56.48 µM), Huh-7 (IC_50_ = 53.95 µM), and Hepa1-6 (IC_50_ = 404.1 µM) cell lines were exposed to varying concentrations of CKD-581 for 24 hours, resulting in inhibited growth and proliferation. The antitumor effect was found to increase with the extension of treatment to 48 and 72 hours (Figures 6A to 6C). Figures 6D to 6F show that CKD-581 inhibited the migration and invasion capabilities of these LIHC cell lines, as confirmed by the dramatically reduced number of cells that moved to the lower surface of the chamber in the Transwell assay. Figure 6G shows that CKD-581 inhibited LIHC cell colony growth in a concentration-dependent way in the 14-day plate clone experiment. The use of flow cytometry revealed an augmented cell death rate in CKD-581-treated hepatocellular carcinoma cells (Figures 6H to 6J). Taken together, our results indicate that LIHC cell proliferation, migration, and invasion are inhibited and apoptosis is enhanced when CKD-581 inhibits endogenous HDAC expression.

### Endogenous HDAC expression inhibition inhibits tumor growth in mice *in vivo*

**Figure 7:**
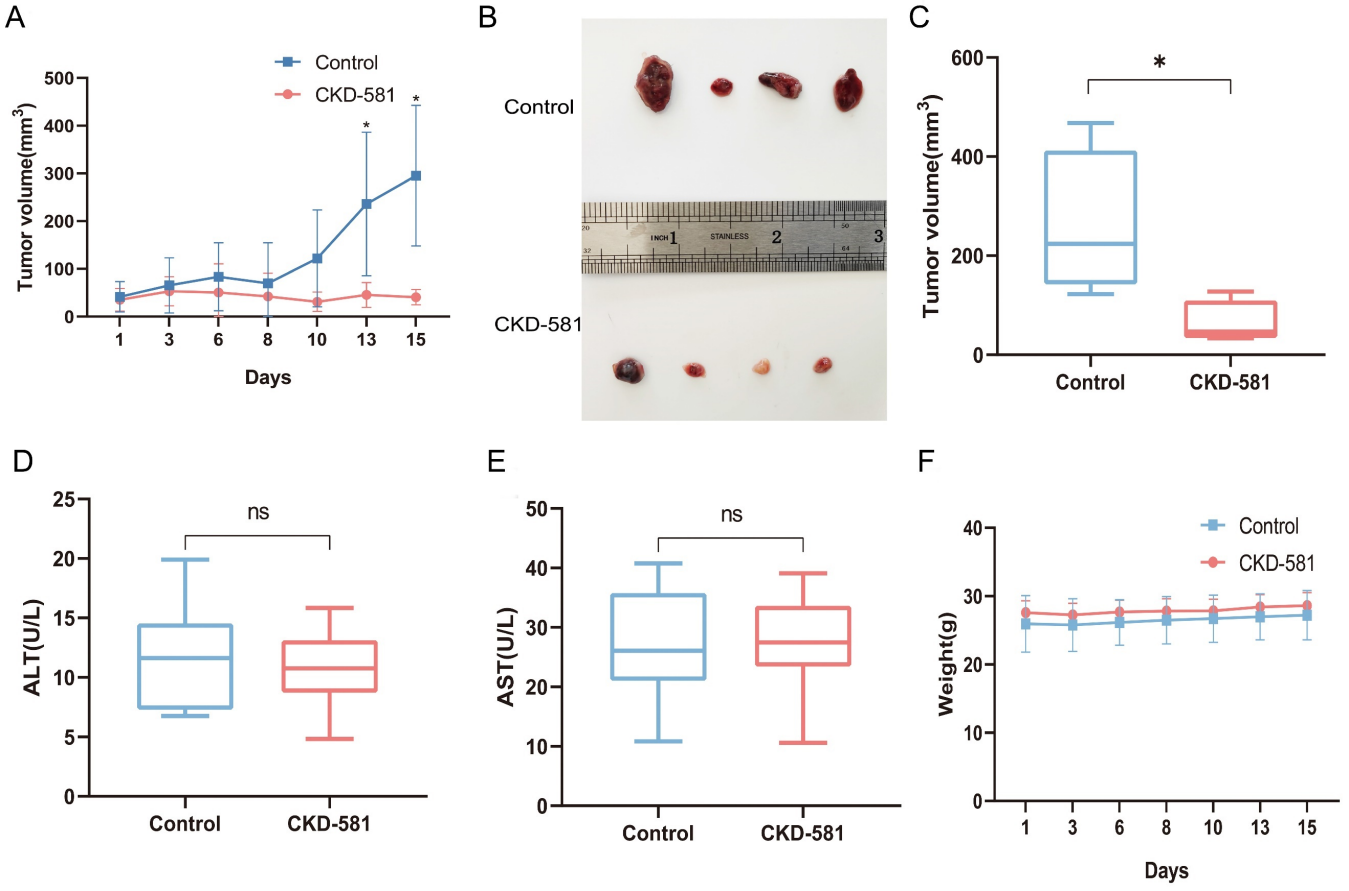
Inhibition of tumor growth by CKD-581 *in vivo*. (A) Changes in tumor volume over the treatment period in control and treated mice. (B) Large tumor specimens from mice at the end of the treatment period. (C) Tumor volumes in the control and treatment groups at the end of the treatment period. (D) Serum ALT and (E) AST levels in mice. (F) Changes in body weight of mice over the treatment period. ns, not significant; *P < 0.05

Following the *in vitro* experiments, the anti-tumor efficacy of CKD-581 was assessed *in vivo* in mice. In the treatment group, notable anti-tumor effects were observed starting from day 13 post-administration (Figure 7A). The tumor volume of the treatment group was considerably less than that of the control group after two weeks of treatment (*P* < 0.05) (Figures 7B and 7C). Assessment of CKD-581's hepatic safety was done by measuring ALT and AST levels in urine. The levels of ALT and AST did not differ significantly between the treatment and control groups, as shown in Figures 7D and 7E. Furthermore, there was no discernible variation in total body mass index (Figure 7F) between the two sets of data.

### CKD-581 exerts anti-tumor effects by regulating the cell cycle

**Figure 8:**
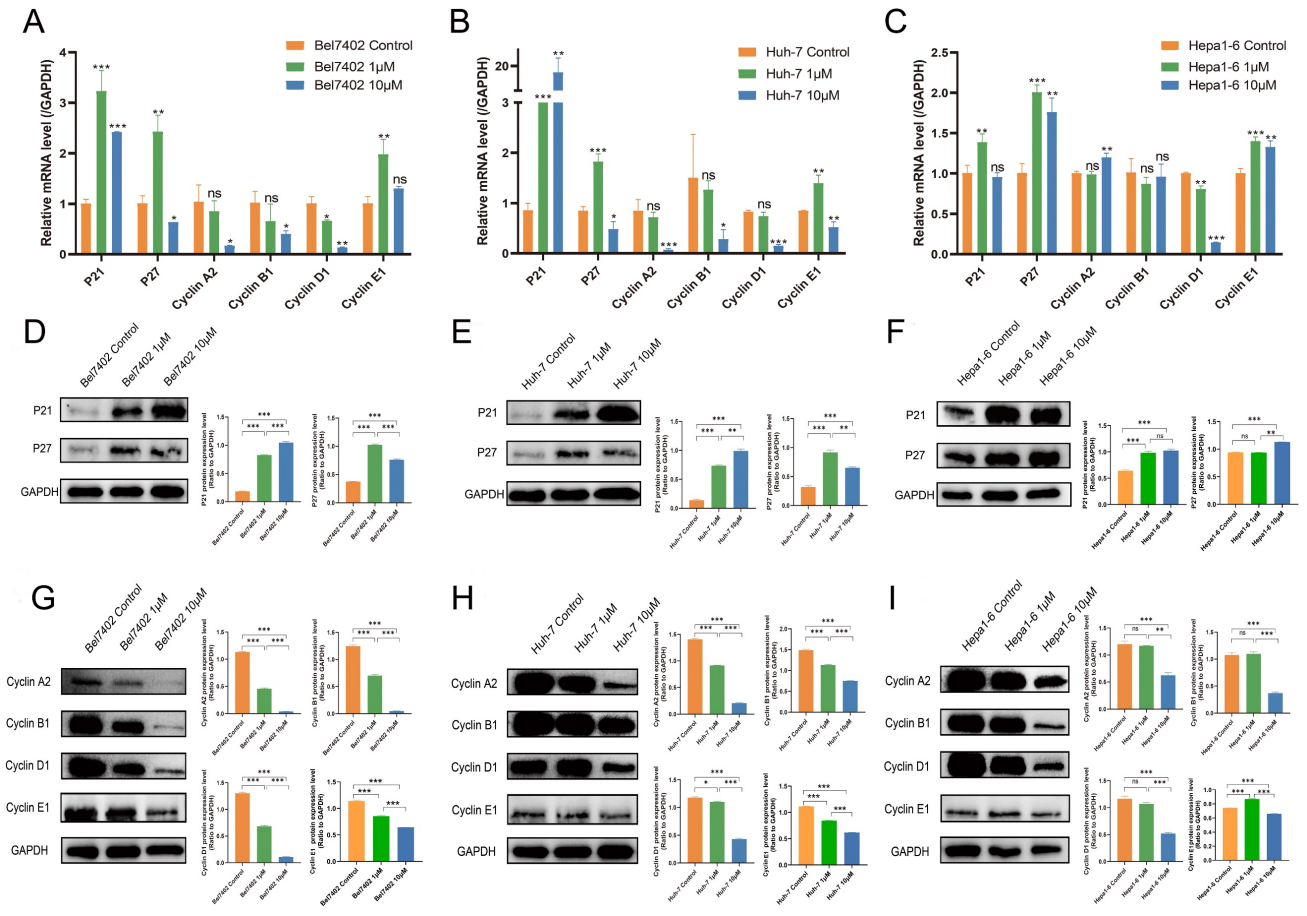
CKD-581 modulates anti-tumor effects by regulating the cell cycle. (A-C) Effects of various concentrations of CKD-581 on mRNA expression levels of cyclin proteins, P21, and P27 in Bel7402, Huh-7, and Hepa1-6 cell lines. (D-I) Impact of various concentrations of CKD-581 on protein expression levels of P21, P27, and cyclins A2, B1, D1, and E1 in these cell lines. (K) Expression and subcellular localization of P21, P27, cyclins A2, B1, D1, and E1 in Bel7402, Huh-7, and Hepa1-6 cell lines following CKD-581 treatment. LIHC, liver hepatocellular carcinoma. ns, not significant; *P < 0.05, **P < 0.01, ***P < 0.001

**Figure 9:**
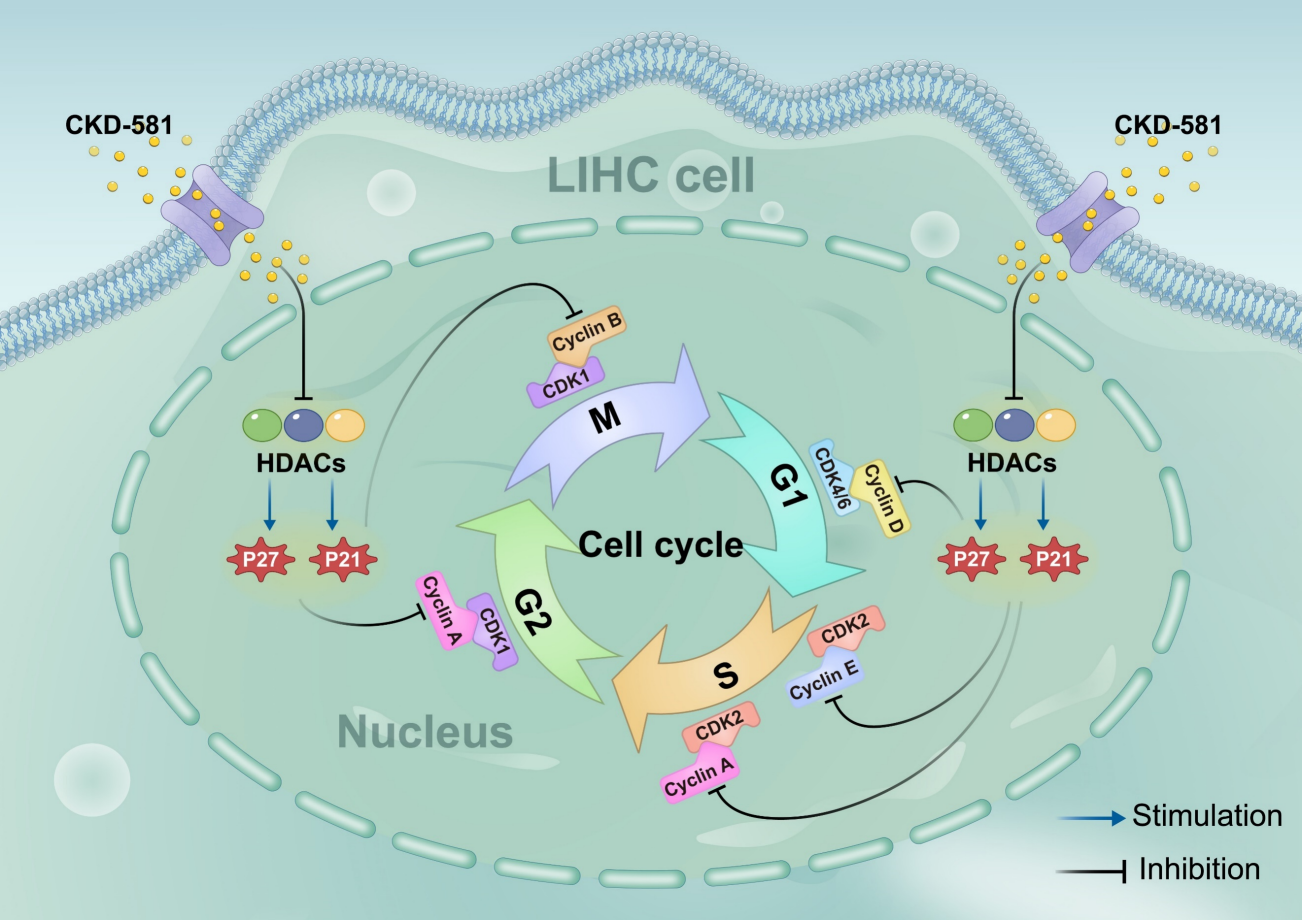
Schematic diagram of the hypothesis that CKD-581 inhibits HDACs, thereby inducing cell cycle arrest in LIHC cells. LIHC, liver hepatocellular carcinoma.

Based on KEGG enrichment analysis results, the impact of CKD-581 on cell cycle-related genes/proteins in LIHC was investigated. In LIHC cells, CKD-581 was observed to decrease cyclin A2, B1, and D1 mRNA levels while increasing P21 and P27 mRNA levels (Figures 8A to 8C). Immunoblotting revealed that in LIHC cells, P21 and P27 protein expression was up after 24 hours of treatment with CKD-581 (1 µM or 10 µM), but cyclins A2, B1, D1, and E1 expression was decreased (Figures 8D to 8I). Furthermore, it was observed that these cell cycle regulatory proteins were predominantly located in the cytoplasm and highly expressed in the nuclei of mitotically active cells, supporting their vital roles in cell proliferation (Supplementary Figures 1-6). These findings suggest that CKD-581 may exert its anticancer effects through modulation of the cell cycle, as illustrated in Figure 9.

## Discussion

Research has demonstrated that the HDAC family of genes is involved in tumor growth and drug resistance. This includes chemoresistance and anti-apoptosis mechanisms 20. By hyperacetylating substrates that are not histones but histone-related, HDACs bring about a wide range of cellular and molecular consequences. They also promote tumor growth by changing important molecules to influence oncogenic cell signaling pathways or by suppressing tumor suppressor gene expression 21. There has been a lack of investigation into the function of HDAC in LIHC.

Understanding the biological roles and possible molecular pathways of endogenous HDACs, as well as their prognostic value in LIHC, were the objectives of this investigation. A risk score prognostic model was constructed using six genes (*HDAC1*, *HDAC4*, *HDAC5*, *HDAC11*, *SIRT6*, and *SIRT7*), selected from a pool of 18 HDAC genes strongly associated with LIHC. Patients were classified as high-risk or low-risk based on the median values of the risk score. The high-risk group had a significantly lower OS when clinical staging was higher. Multiple independent prognostic studies have shown that the risk score is an independent prognostic factor. Based on the ROC curves, the risk score has a good predictive accuracy for LIHC and can be used as a prognostic marker. The 1-, 2-, and 3-year OS AUCs were 0.710, 0.679, and 0.661, respectively.

The risk model was constructed based on six HDAC genes: *HDAC1*, *HDAC4*, *HDAC5*, *HDAC11*, *SIRT6*, and *SIRT7*, all of which were upregulated in LIHC tissues. It has been demonstrated that the absence or downregulation of HDAC1 leads to cell cycle arrest at the G1 phase or G2/M transition, thereby inhibiting cell growth and increasing the percentage of apoptotic cells 22. High HDAC1 expression in LIHC patients, on the other hand, is linked to worse tissue differentiation, more advanced tumor lymph node metastatic staging, lower survival rates, and an increased incidence of cancer cells invading the portal vein 23. These findings align with the outcomes of our study. Inhibition of HDAC4 enhances the cytotoxic effects of cisplatin and impedes tumor cell growth 24. Similarly, downregulation of HDAC5, like HDAC1, triggers a G1 phase block in the cell cycle and promotes apoptosis 25. The presence of HDAC5, which is abundant in cancerous epithelial cells' cytoplasm, is positively associated with lymph node and distant metastasis 26, 27. A high correlation between HDAC11 and tumor development, microvascular invasion, tumor differentiation, and clinical staging in patients with LIHC suggests that it may have a regulatory role in maintaining cancer stemness 28. In LIHC cells, SIRT6 has been found to promote cell migration, invasion, and epithelial-mesenchymal transition 29. Additionally, it plays a role in supporting tumor development by protecting against DNA damage and cellular senescence 30. Reducing SIRT7 levels causes cell cycle arrest and slows cell growth 31. By blocking SIRT7, adriamycin-induced P53 activation is amplified in mice xenografts, leading to apoptosis and tumor growth suppression 32. These results substantiate the high clinical utility of our risk score prognostic model based on these genes.

Our investigation into the correlation between risk score and immune infiltration revealed that the high-risk group exhibited reduced numbers of NK cells and neutrophils. The innate immune system's cytotoxic lymphocytes known for their ability to kill cancer cells, known as NK cells, were shown to be correlated with a decline in anti-tumor immunity in high-risk LIHC patients 33. Neutrophils, which participate in various carcinogenic stages including tumor initiation, growth, proliferation, and metastasis 34, were unexpectedly found in lower proportions in the high-risk group, suggesting a disruption in the tumor microenvironment, though the exact cause requires further investigation. Furthermore, a reduced type II IFN response—which normally aids in host defense and immunological surveillance and promotes tumor cell apoptosis—was linked to a high-risk score 35. C1 was associated with high-risk scores according to immunophenotype analysis, but C3 and C4 were associated with low-risk scores; C1 is conducive to tumor development, whereas C3 and C4 act as effective protective factors. This aligns with previous findings that higher immunophenotypes, indicative of increased cytotoxic cells, are associated with better patient survival 36.

Furthermore, we analyzed the expression differences of 6 HDACs in LIHC tissues compared to adjacent normal tissues and observed that five HDACs, with the exception of HDAC5, were upregulated in LIHC. Patients whose HDAC1 expression was high had a much worse survival rate compared to those whose expression was low, according to survival analysis focusing on HDAC1. Univariate and multivariate Cox analyses showed that patients with LIHC with a high level of HDAC1 expression had a poorer prognosis. The strong correlation between HDAC1 mRNA expression and tumor size further supports its central role in the preconstructed prognostic model.

There are currently four HDAC inhibitors that have been approved by the US Food and Drug Administration for cancer treatment 37. However, their effectiveness in treating solid tumors has been limited. Our study demonstrates that CKD-581, a novel HDAC inhibitor, exhibits strong antitumor activity against LIHC cells both *in vivo* and *in vitro*. CKD-581 has been demonstrated to effectively hinder the growth, proliferation, migration, and invasion of LIHC cell lines, as well as induce apoptosis at certain doses, according to biological function experiments. *In vivo,* CKD-581 not only reduces tumor size in mice but also displays potent anti-tumor effects without significant hepatotoxic or adverse reactions, suggesting a favorable safety profile for CKD-581.

Based on KEGG enrichment analysis results, we explored how HDAC inhibitors mediate their antitumor effects. By reducing cyclin A2, B1, and D1 expression and increasing P21 and P27 expression, CKD-581 was discovered to suppress LIHC cell growth. There are four separate phases in the cell cycle: G1, S, G2, and M 38. Cell cycle protein-dependent kinases (CDKs), cyclins, and CDK inhibitors (CDKIs) are all involved in cell cycle regulation. CDK activation happens upon binding to a cyclin 39. Cell cycle progression relies on cyclins and CDKs (1, 2, 4, and 6). Cell cycle protein D, the first to respond to mitotic signals, activates CDK4 and CDK6 during the G1 phase, acting as a growth factor sensor 40. CDK2 activation by cell cycle protein E1 facilitates the G1 to S phase transition. In the S phase, cell cycle protein A interacts with CDK1/2 to promote transition to the M phase. By the end of the G2 phase, CDK1 associates with cyclin B. Uncontrolled CDK activation can lead to continuous cell division, contributing to tumor development; this activity is regulated by CDKIs, such as the CIP/KIP family (P21, P27, P57) and the INK family (P15, P16, P18, P19) 41. CIP/KIP family members inhibit the activity of A, B, D, and E/CDK complexes, leading to cell cycle arrest. P21, a widely recognized cell cycle inhibitor, suppresses tumor growth by causing G1 phase arrest 42. Normally, P27 levels are strictly regulated, and its upregulation prevents cyclin binding to CDK, thus blocking the G1 to S phase transition 43. Low levels of P21 and P27, which act as tumor suppressors, can lead to unrestrained cell division and proliferation. In this study, CKD-581 was shown to block the G1/S phase by markedly upregulating P21 and P27 and downregulating cyclins A2, B1, and D1, thereby inhibiting cell proliferation and promoting cell death in LIHC cells (Figure 9). This finding supports our KEGG analysis results.

To summarize, this study examined the expression and prognostic significance of HDAC genes in LIHC, establishing a model based on six HDACs (*HDAC1*, *HDAC4*, *HDAC5*, *HDAC11*, *SIRT6*, and *SIRT7*). This model proved effective in predicting OS in LIHC patients, with HDAC1 identified as a key component. Additionally, CKD-581, an HDAC inhibitor, was shown to exert antitumor effects by regulating the cell cycle. Despite its insights, this study is limited by the absence of experiments on exogenous HDAC genes to confirm the cellular functions attributed to HDACs. Moreover, the enrollment of limited number of LIHC cases in the ICGC database resulted in the non-significant OS prediction in the validation cohort. Finally, the TCGA test set and the ICGC validation set arises due to the clinical grading information is absent in the ICGC database, hence lacking related analyses and resulting in weak correlation results in the ICGC validation set. These limitations are intended to be addressed in future research.

## Supplementary Material

Supplementary figures.

## Figures and Tables

**Table 1:** Primer sequences.

Name	Sequence (5'→3')	mer	OD/Tube	nmol/Tube	MW	GC%	Tm(°C)
HDAC1(M)-F	TGCGTTCTATTCGCCCAGATA	21	1	5.3	6372.1	47.6	58.0
HDAC1(M)-R	CCTCCCGTGGACAACTGAC	19	1	5.8	5733.7	63.2	61.9
HDAC4(M)-F	CACTGCATTTCCAGCGATCC	20	1	5.7	6012.9	55.0	59.9
HDAC4(M)-R	AAGACGGGGTGGTTGTAGGA	20	1	4.7	6302.1	55.0	59.9
HDAC5(M)-F	AGCACCGAGGTAAAGCTGAG	20	1	4.7	6200.1	55.0	59.9
HDAC5(M)-R	GAACTCTGGTCCAAAGAAGCG	21	1	4.7	6464.2	52.4	60.0
HDAC11(M)-F	CTGTGCCTATGCAGACATCAC	21	1	5.2	6366.1	52.4	60.0
HDAC11(M)-R	GTGGCGGTTGTAAACATCCAT	21	1	4.9	6461.2	47.6	58.0
SIRT6(M)-F	CTCCAGCGTGGTTTTCCACA	20	1	5.7	6043.9	55.0	59.9
SIRT6(M)-R	GCCCATGCGTTCTAGCTGA	19	1	5.8	5779.8	57.9	59.7
SIRT7(M)-F	GCACTTGGTTGTCTACACGG	20	1	5.5	6124.0	55.0	59.9
SIRT7(M)-R	TGTCCATACTCCATTAGGACCC	22	1	5.1	6630.3	50.0	60.1
GAPDH(M)-F	AATGGATTTGGACGCATTGGT	21	1	4.8	6516.3	42.9	56.1
GAPDH(M)-R	TTTGCACTGGTACGTGTTGAT	21	1	5.2	6458.2	42.9	56.1
P21(H)-F	CGATGGAACTTCGACTTTGTCA	22	1	4.8	6725.4	45.5	58.2
P21(H)-R	GCACAAGGGTACAAGACAGTG	21	1	4.4	6513.3	52.4	60.0
P27(H)-F	AACGTGCGAGTGTCTAACGG	20	1	5.0	6182.0	55.0	59.9
P27(H)-R	CCCTCTAGGGGTTTGTGATTCT	22	1	5.2	6723.3	50.0	60.1
Cyclin A2(H)-F	GGATGGTAGTTTTGAGTCACCAC	23	1	4.5	7094.6	47.8	60.2
Cyclin A2(H)-R	CACGAGGATAGCTCTCATACTGT	23	1	4.6	7023.6	47.8	60.2
Cyclin B1(H)-F	TTGGGGACATTGGTAACAAAGTC	23	1	4.2	7127.7	43.5	58.4
Cyclin B1(H)-R	ATAGGCTCAGGCGAAAGTTTTT	22	1	4.6	6789.4	40.9	56.3
Cyclin D1(H)-F	CAATGACCCCGCACGATTTC	20	1	5.5	6021.9	55.0	59.9
Cyclin D1(H)-R	CATGGAGGGCGGATTGGAA	19	1	5.0	5957.9	57.9	59.7
Cyclin E1(H)-F	GCCAGCCTTGGGACAATAATG	21	1	4.8	6455.2	52.4	60.0
Cyclin E1(H)-R	CTTGCACGTTGAGTTTGGGT	20	1	5.5	6170.0	50.0	57.8
P21(M)-F	CGAGAACGGTGGAACTTTGAC	21	1	4.7	6495.3	52.4	60.0
P21(M)-R	CCAGGGCTCAGGTAGACCTT	20	1	5.3	6118.0	60.0	61.9
P27(M)-F	TCAAACGTGAGAGTGTCTAACG	22	1	4.5	6783.4	45.5	58.2
P27(M)-R	CCGGGCCGAAGAGATTTCTG	20	1	5.2	6158.0	60.0	61.9
Cyclin A2(M)-F	GCCTTCACCATTCATGTGGAT	21	1	5.3	6372.1	47.6	58.0
Cyclin A2(M)-R	TTGCTCCGGGTAAAGAGACAG	21	1	4.7	6495.3	52.4	60.0
Cyclin B1(M)-F	TCGTTCACCAGCGATCTGTC	20	1	5.7	6043.9	55.0	59.9
Cyclin B1(M)-R	CGAAGCCCTGCCAATACCATA	21	1	5.0	6344.2	52.4	60.0
Cyclin D1(M)-F	GCGTACCCTGACACCAATCTC	21	1	5.3	6311.1	57.1	61.9
Cyclin D1(M)-R	ACTTGAAGTAAGATACGGAGGGC	23	1	4.1	7161.7	47.8	60.2
Cyclin E1(M)-F	GAAAAGCGAGGATAGCAGTCAG	22	1	4.1	6866.5	50.0	60.1
Cyclin E1(M)-R	CCCAATTCAAGACGGGAAGTG	21	1	4.7	6464.2	52.4	60.0

**Table 2:** Differentially expressed genes in LIHC.

Gene	conMean	treatMean	logFC	pValue	fdr
HDAC1	10.14285	20.77175384	1.03416	5.37E-19	1.01E-18
HDAC4	0.247534	0.787871827	1.670334	5.63E-23	2.39E-22
HDAC5	3.517294	8.262054521	1.232035	5.92E-22	1.68E-21
HDAC7	1.132222	3.061601049	1.435129	4.44E-15	6.86E-15
HDAC8	0.18237	0.377620457	1.050068	5.57E-22	1.68E-21
HDAC10	0.051058	0.166399178	1.704439	4.77E-21	1.16E-20
HDAC11	0.930702	4.98238694	2.420446	1.83E-27	3.11E-26
SIRT4	1.093688	2.267897671	1.052154	7.13E-16	1.21E-15
SIRT6	2.532166	6.132379268	1.276075	1.88E-23	1.07E-22
SIRT7	1.171316	3.695575534	1.657669	7.68E-27	6.53E-26

**Table 3:** Univariate independent prognostic analysis.

Id	HR	95% Low	95% High	P value
HDAC1	1.9506136	1.47785493	2.574605486	2.38E-06
HDAC2	2.2413876	1.654272032	3.03687559	1.91E-07
HDAC4	2.04100885	1.314764087	3.168414127	0.001475036
HDAC5	1.5724753	1.1760063	2.102606566	0.002260432
HDAC7	1.27370289	1.010233712	1.605884883	0.040749063
HDAC10	3.26220209	1.016148118	10.47284573	0.046935458
HDAC11	1.48783175	1.200947157	1.843247886	0.000277569
SIRT6	1.56739156	1.179448881	2.082935808	0.001951356
SIRT7	1.54247809	1.166652403	2.039372352	0.002351649

## Abbreviations

ALT: alanine aminotransferase
AST: aspartate aminotransferase
ANOVA: one-way analysis of variance
CNV: copy number variation
DNAss: DNA stem cell scores
GSEA: gene set enrichment analysis
HDACs: histone deacetylases
ICGC: International Cancer Genome Consortium
IFN: interferon
LASSO: least absolute shrinkage and selection operator
LIHC: liver hepatocellular carcinoma
NK: natural killer
OS: overall survival
TCGA: The Cancer Genome Atlas
t-SNE: t-distributed stochastic neighbor embedding
RT-qPCR: Real-time quantitative PCR
RNAss: RNA stem cell scores
SDS-PAGE: sodium dodecyl sulfate-polyacrylamide gels electrophoresis
SD: standard deviation
ssGSEA: single sample gene set enrichment analysis
UCSC: University of California Santa Cruz
