# Supplementary material for: Comprehensive Analysis of the Prognostic Implications and Biological Function of HDACs in Liver Hepatocellular Carcinoma
Source: Int J Med Sci. 2024 Oct 28;21(14):2807–23. doi: 10.7150/ijms.97169 (PMC11539383; doi:10.7150/ijms.97169)
Supplement: Supplementary file 1 — Supplementary figures. [file ijmsv21p2807s1.pdf]

Supplementary Figures. Expressions of cyclin A2 (Supplementary Figure 1), cyclin B1 (Supplementary Figure 2), cyclin D1 (Supplementary Figure 3), cyclin E1 (Supplementary Figure 4), P21 (Supplementary Figure 5), and P27 (Supplementary Figure 6) in Bel7402, Huh-7, and Hepa1-6 cell lines detected using indirect immunofluorescence assay. Nuclei were stained with blue fluorescence by DAPI, and target proteins were labeled with red-fluorescence by Cy3.

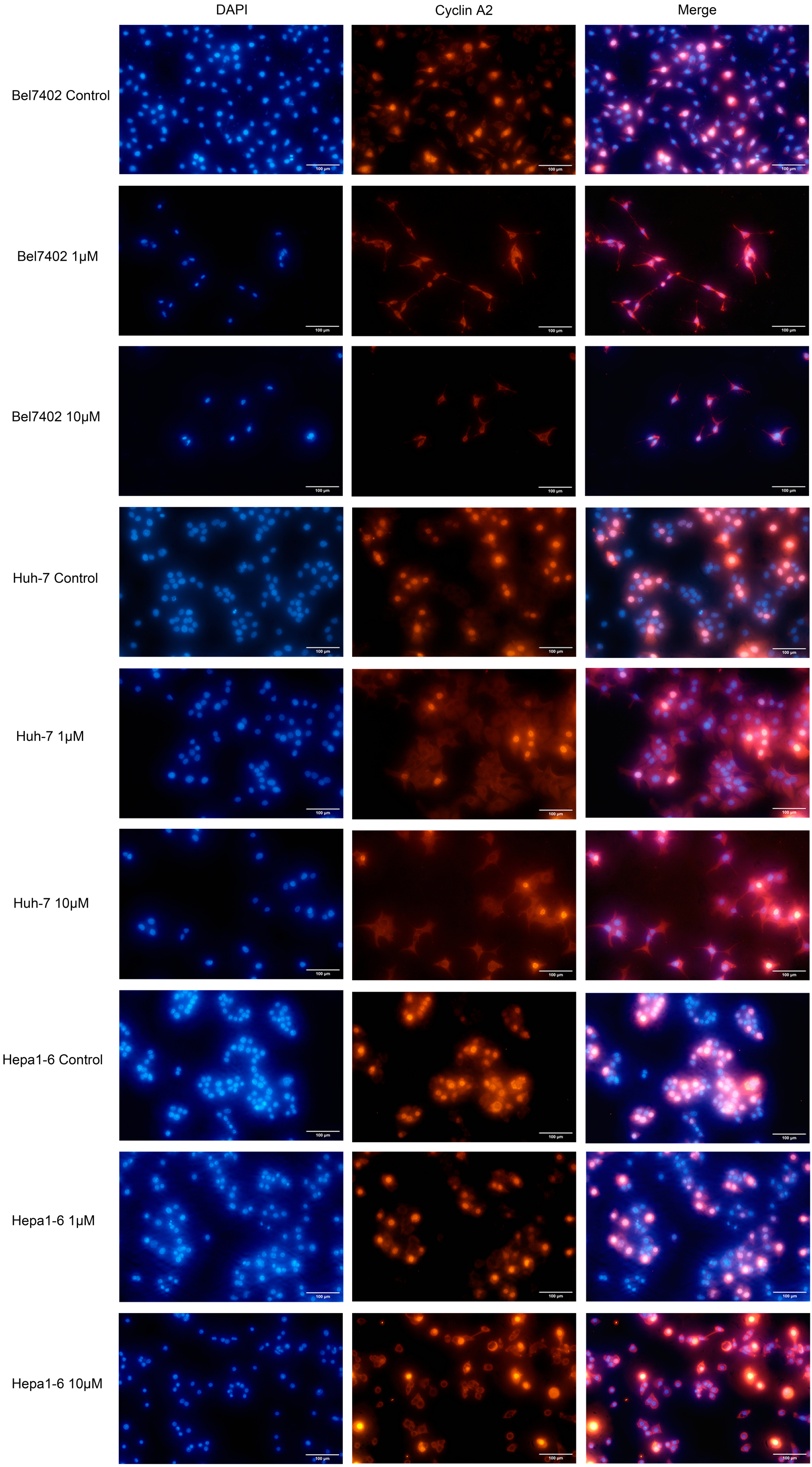

DAPI
Cyclin A2
Merge
Bel7402 Control
Bel7402 1μM
Bel7402 10μM
Huh-7 Control
Huh-7 1μM
Huh-7 10μM
Hepa1-6 Control
Hepa1-6 1μM
Hepa1-6 10μM
100 μm

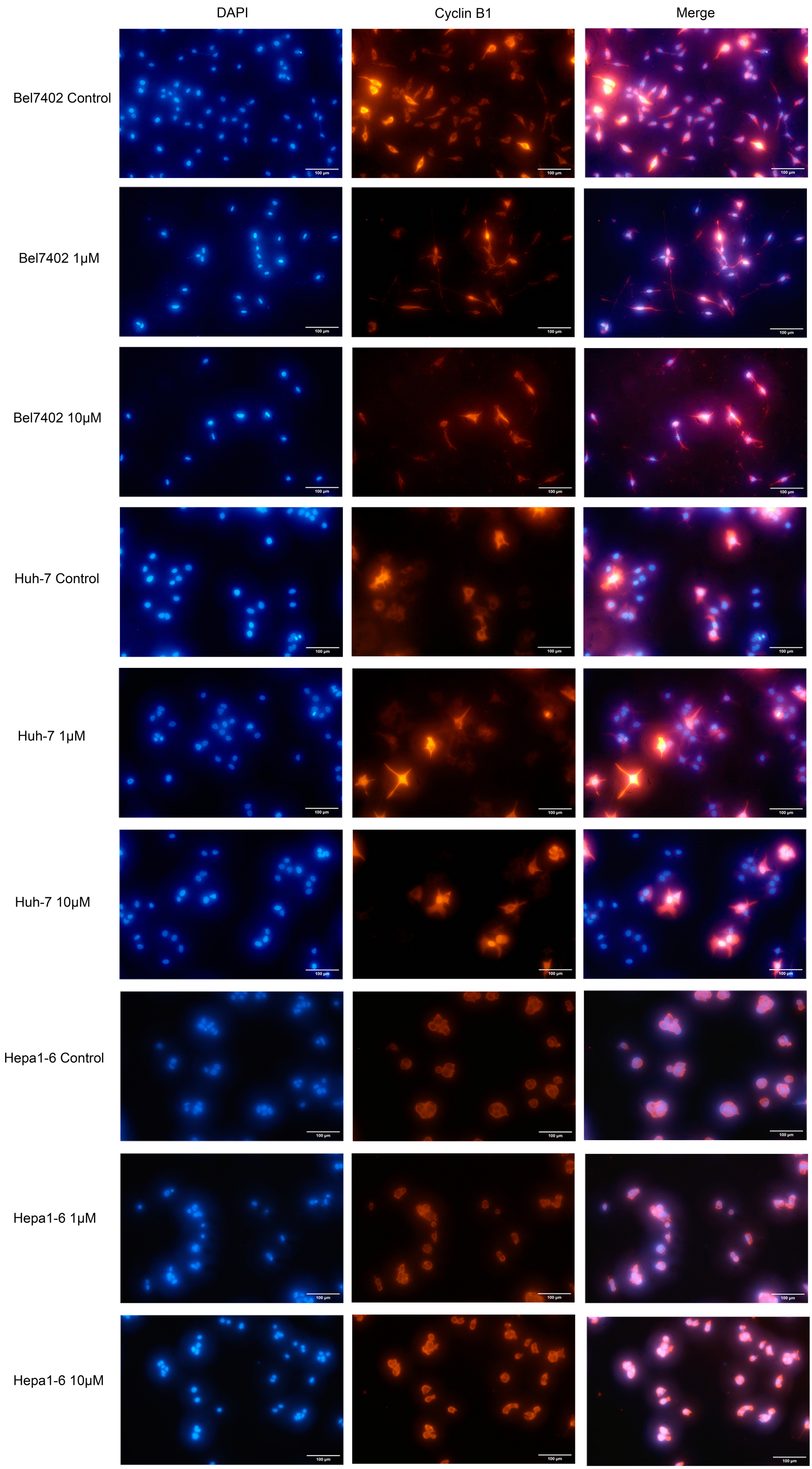
DAPI
Cyclin B1
Merge
Bel7402 Control
Bel7402 1μM
Bel7402 10μM
Huh-7 Control
Huh-7 1μM
Huh-7 10μM
Hepa1-6 Control
Hepa1-6 1μM
Hepa1-6 10μM

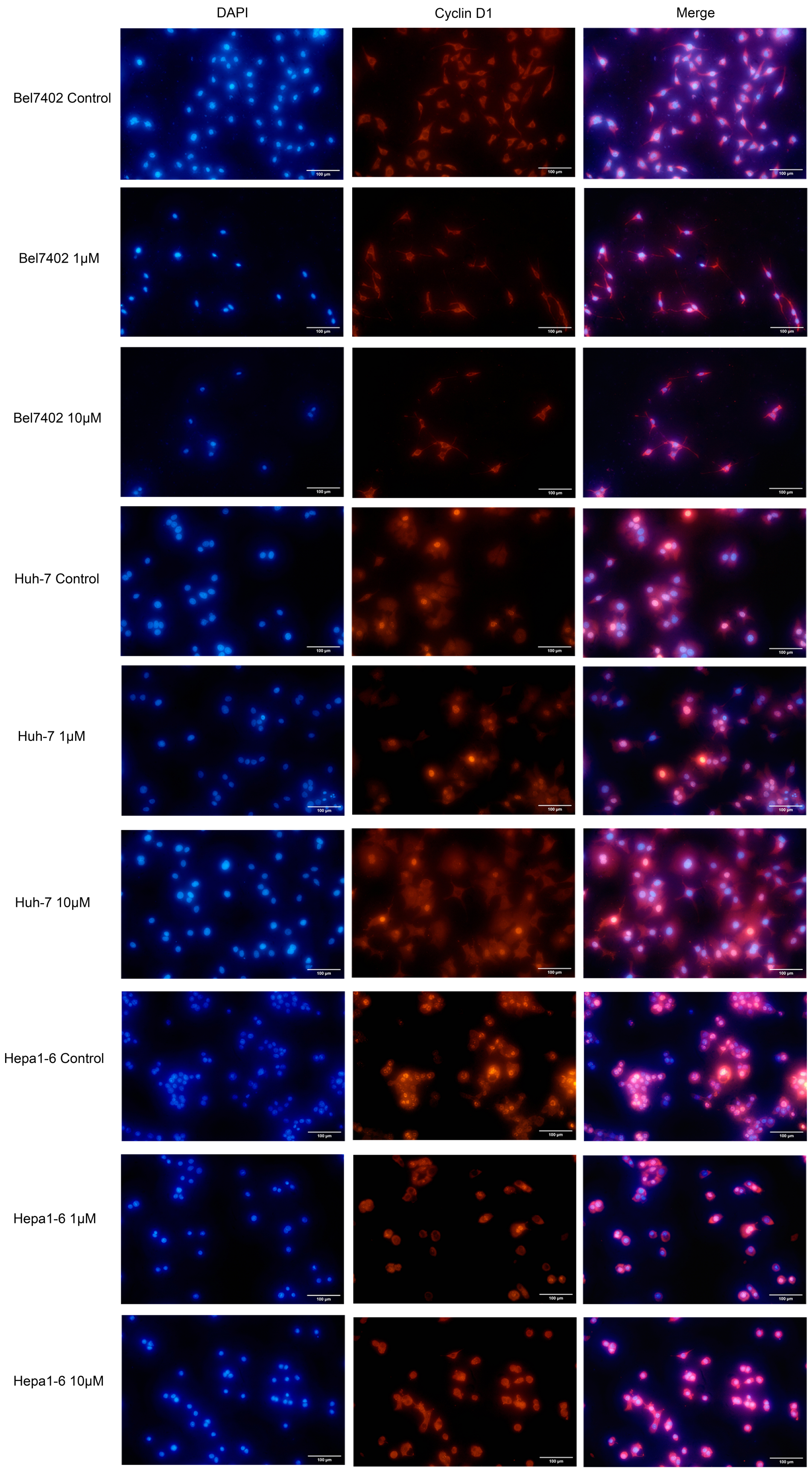
DAPI
Cyclin D1
Merge
Bel7402 Control
Bel7402 1μM
Bel7402 10μM
Huh-7 Control
Huh-7 1μM
Huh-7 10μM
Hepa1-6 Control
Hepa1-6 1μM
Hepa1-6 10μM

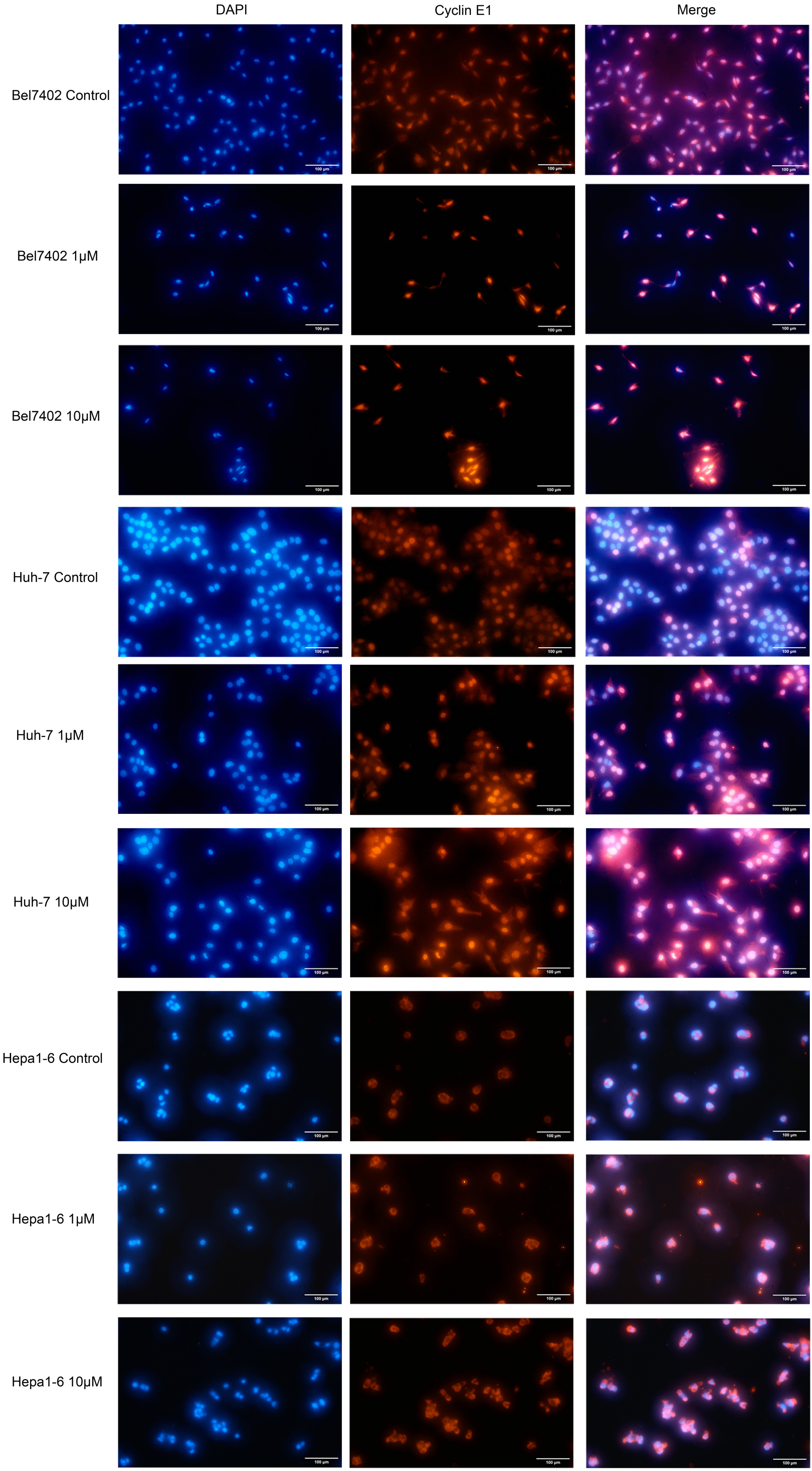
DAPI
Cyclin E1
Merge
Bel7402 Control
Bel7402 1μM
Bel7402 10μM
Huh-7 Control
Huh-7 1μM
Huh-7 10μM
Hepa1-6 Control
Hepa1-6 1μM
Hepa1-6 10μM
100 μm

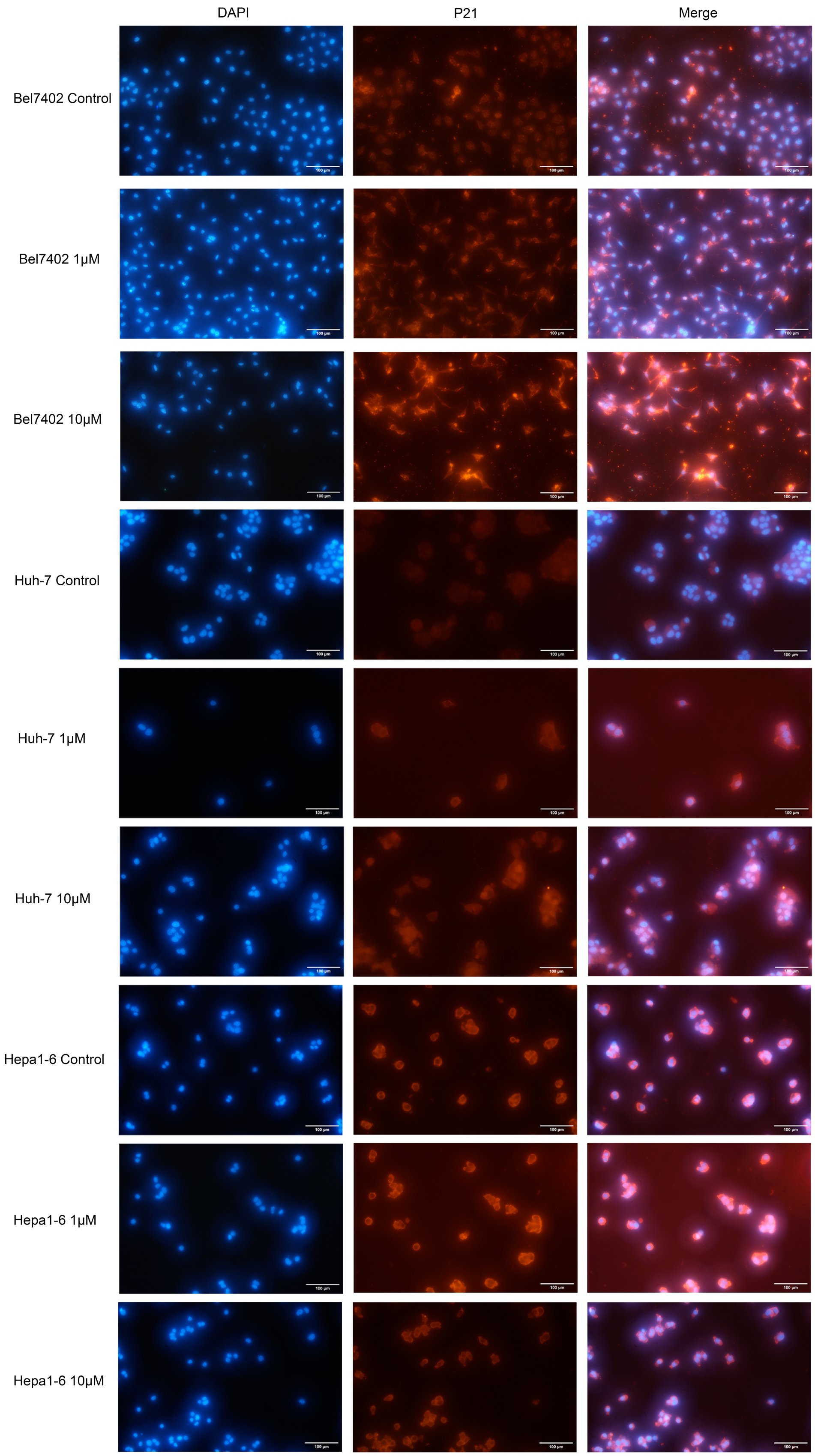
DAPI
P21
Merge
Bel7402 Control
Bel7402 1μM
Bel7402 10μM
Huh-7 Control
Huh-7 1μM
Huh-7 10μM
Hepa1-6 Control
Hepa1-6 1μM
Hepa1-6 10μM

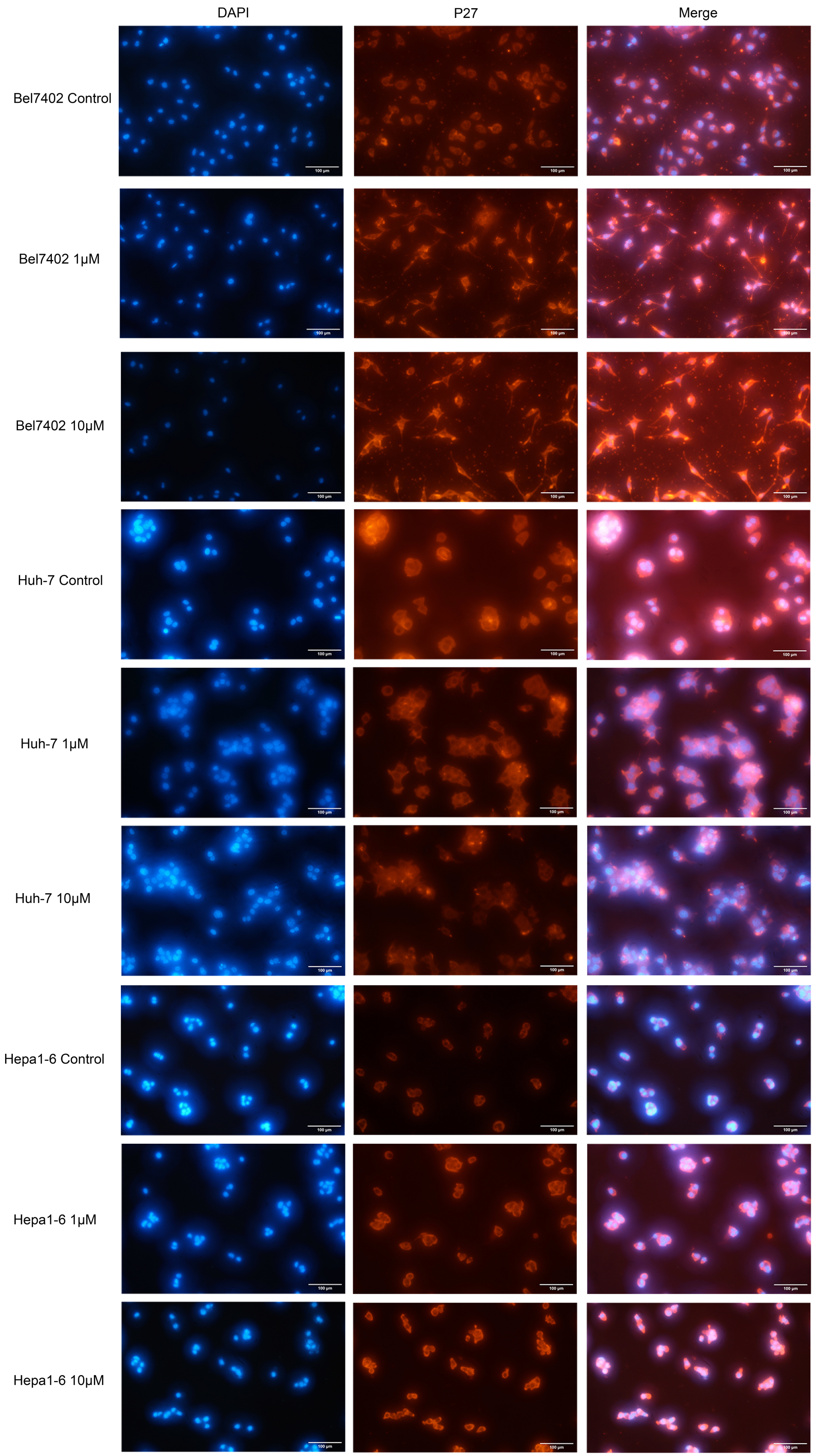

DAPI
P27
Merge
Bel7402 Control
Bel7402 1μM
Bel7402 10μM
Huh-7 Control
Huh-7 1μM
Huh-7 10μM
Hepa1-6 Control
Hepa1-6 1μM
Hepa1-6 10μM
100 μm
